# Angle-stable interlocking nailing in a canine critical-sized femoral defect model for bone regeneration studies: In pursuit of the principle of the 3R’s

**DOI:** 10.3389/fbioe.2022.921486

**Published:** 2022-09-02

**Authors:** W. B. Saunders, L. M. Dejardin, E. V. Soltys-Niemann, C. N. Kaulfus, B. M. Eichelberger, L. K. Dobson, B. R. Weeks, S. C. Kerwin, C. A. Gregory

**Affiliations:** ^1^ Department of Small Animal Clinical Sciences, College of Veterinary Medicine and Biomedical Sciences, Texas A & M University, College Station, TX, United States; ^2^ Department of Small Animal Clinical Sciences, College of Veterinary Medicine, Michigan State University, East Lansing, MI, United States; ^3^ Department of Veterinary Pathobiology, College of Veterinary Medicine and Biomedical Sciences, Texas A & M University, College Station, TX, United States; ^4^ Department of Large Animal Clinical Sciences, College of Veterinary Medicine and Biomedical Sciences, Texas A & M University, College Station, TX, United States; ^5^ Department of Molecular and Cellular Medicine, Institute for Regenerative Medicine, School of Medicine, Texas A & M Health Science Center, College Station, TX, United States

**Keywords:** canine, large animal model, critical-sized defect, non-union, bone regeneration, interlocking nail, biomechanics, computed tomography

## Abstract

**Introduction:** Critical-sized long bone defects represent a major therapeutic challenge and current treatment strategies are not without complication. Tissue engineering holds much promise for these debilitating injuries; however, these strategies often fail to successfully translate from rodent studies to the clinical setting. The dog represents a strong model for translational orthopedic studies, however such studies should be optimized in pursuit of the Principle of the 3R’s of animal research (replace, reduce, refine). The objective of this study was to refine a canine critical-sized femoral defect model using an angle-stable interlocking nail (AS-ILN) and reduce total animal numbers by performing imaging, biomechanics, and histology on the same cohort of dogs.

**Methods:** Six skeletally mature hounds underwent a 4 cm mid-diaphyseal femoral ostectomy followed by stabilization with an AS-ILN. Dogs were assigned to autograft (*n* = 3) or negative control (*n* = 3) treatment groups. At 6, 12, and 18 weeks, healing was quantified by ordinal radiographic scoring and quantified CT. After euthanasia, femurs from the autograft group were mechanically evaluated using an established torsional loading protocol. Femurs were subsequently assessed histologically.

**Results:** Surgery was performed without complication and the AS-ILN provided appropriate fixation for the duration of the study. Dogs assigned to the autograft group achieved radiographic union by 12 weeks, whereas the negative control group experienced non-union. At 18 weeks, median bone and soft tissue callus volume were 9,001 mm^3^ (range: 4,939–10,061) for the autograft group and 3,469 mm^3^ (range: 3,085–3,854) for the negative control group. Median torsional stiffness for the operated, autograft treatment group was 0.19 Nm/° (range: 0.19–1.67) and torque at failure was 12.0 Nm (range: 1.7–14.0). Histologically, callus formation and associated endochondral ossification were identified in the autograft treatment group, whereas fibrovascular tissue occupied the critical-sized defect in negative controls.

**Conclusion:** In a canine critical-sized defect model, the AS-ILN and described outcome measures allowed refinement and reduction consistent with the Principle of the 3R’s of ethical animal research. This model is well-suited for future canine translational bone tissue engineering studies.

## Introduction

Critical-sized long bone defects are most commonly caused by vehicular trauma, gunshot or blast injuries, infection, tumor resection, and developmental deformities (Roddy et al., 2017). Critical-sized defects are at least two times the diameter of the affected long bone and represent a major therapeutic challenge (Roddy et al., 2017; Schemitsch, 2017). Current treatment strategies to “bridge the gap” include mechanical stabilization followed by autologous bone grafting, distraction osteogenesis, application of BMPs, or the induced membrane technique (Roddy et al., 2017). Unfortunately, none of these treatments are uniformly effective and each is associated with its own complications. Therefore, substantial effort has been devoted to the development of tissue engineering solutions to improve clinical success (Muschler et al., 2010; Roddy et al., 2017; Manzini et al., 2021; Mohaghegh et al., 2021).

Translating promising tissue engineering solutions from *in vitro* and rodent proof of concept studies to clinical use is fraught with challenges (Reichert et al., 2010; Sastry, 2014; Zheng et al., 2017). Although laboratory animal models represent an important step in the validation pathway, rodent biology and biomechanics do not simulate the clinical scenarios encountered in clinical orthopedics. For these reasons, large animals such as the sheep, pig, dog, and goat are often used as intermediary, translational models (Arinzeh et al., 2003; Liebschner, 2004; Horner et al., 2010; Muschler et al., 2010; Hahn et al., 2011; Dozza et al., 2018; Ribitsch et al., 2020). The dog represents a strong translational model species for orthopedic tissue engineering (Arinzeh et al., 2003; Lindsey et al., 2006; Hoffman and Dow, 2016). The canine musculoskeletal system experiences similar biomechanical loads to that of humans (Egermann et al., 2005; Pearce et al., 2007). Dogs also exhibit comparable bone development and physiology, complex immune systems and genetics (Ostrander and Giniger, 1997; Ribitsch et al., 2020), and are amenable to assessment tools such as arthroscopy or gait analysis that are used in clinical trials (Neu et al., 2009; Hoffman and Dow, 2016; Feichtenschlager et al., 2018).

Interlocking nails are considered by many to be the standard of care for stabilization of critical-sized bone defects (Rhorer, 2009; Dejardin et al., 2012; Shen et al., 2016; Flagstad et al., 2021; Kang et al., 2021; Viberg et al., 2021). Intramedullary fixation is commonly used for both rodent and large animal critical gap healing models (Lindsey et al., 2006; Schoen et al., 2008; Rhorer, 2009; MSc et al., 2013; Quirk et al., 2016; Shiels et al., 2018; Ozmeric et al., 2020). Despite their widespread use, acute angular instability is an inherent component of traditional interlocking nails and in the clinical setting has been termed “slack” (Lang et al., 1995; Buehler et al., 1997; Gerber and Ganz, 1998; Dejardin et al., 2006, 2014). The result of slack is unpredictably high angular deformation under torsional loads that can lead to delayed or nonunion (Pfeil et al., 2005; Dejardin et al., 2006, 2012, 2014; Lansdowne et al., 2007). In the context of large animal translational studies, slack has the potential to create a biomechanical environment in which implant performance is somewhat variable from animal to animal. One solution for this variability is to increase animal numbers. This, however, increases study costs and raises ethical concerns.

The principles of replacement, reduction, and refinement, known as the Principle of the 3R’s, has guided global ethics of animal research for over 60 years (Kirk, 2018; Angelis et al., 2019). In pursuit of this principle, work has been undertaken to refine the interlocking nail implant to eliminate construct slack by development of an angle-stable interlocking nail (AS-ILN) (Dejardin et al., 2006, 2009; Lansdowne et al., 2007; Ting et al., 2009). This AS-ILN has been used in both induced-injury and clinical canine studies (Dejardin et al., 2014; Marturello et al., 2021). It has definitively been shown to be superior to traditional nails with accelerated healing times, greater callus volume, and improved biomechanical callus properties in a 5 mm, induced-injury, tibial ostectomy model (Dejardin et al., 2014). However, AS-ILNs have not been evaluated in critical-sized defect studies, nor have investigators further reduced animal numbers by performing longitudinal radiographic studies, biomechanics, and histology on the same cohort of animals. Implementation of these strategies would represent an important advancement in pursuit of the Principle of the 3R’s (Kirk, 2018; Angelis et al., 2019).

Therefore, the objective of this study was to optimize a canine critical-sized femoral defect model using an angle-stable interlocking nail (AS-ILN). We hypothesized that: 1) an AS-ILN would provide long term construct stability in a canine femoral critical-sized defect model and 2) the placement of autograft would yield faster and more robust bone regeneration than negative control defects. Radiography, quantitative CT, torsional biomechanics, and histology provided an objective and comprehensive assessment of critical-sized defect healing in a small cohort of dogs. The implant system, surgical technique, and outcome measures described herein are well-suited for future canine translational tissue engineering studies.

## Materials and methods

### Surgical model

This study was performed under the supervision of the Texas A&M University Institutional Animal Care and Use Committee (IACUC) and an approved animal use protocol (AUP 2013–0056). Six, male, skeletally-mature, institutionally-owned hounds were utilized in the study. Age ranged from 2.5 to 6.5 years and body weight from 23.7 to 35.5 kg. Dogs were screened pre-operatively with a general physical exam, detailed orthopedic exam by a board-certified veterinary surgeon as well as complete blood count, serum chemistry, and urinalysis. Prior to surgery, radiographs of the right femur were obtained under sedation with dexmedetomidine (125 μg/m^2^) and butorphanol (0.2 mg/kg), administered intravenously (IV). Sedation was reversed with atipamezole (1,666 μg/m^2^). Dogs were randomly assigned to one of two groups: autograft or negative control. All dogs received a 4 cm mid-diaphyseal femoral ostectomy with resection of periosteum at the defect tissues followed by stabilization with an 8 mm diameter x 172 mm long AS-ILN developed specifically for veterinary use (I-Loc internal fixator, Biomedtrix, Whippany, NJ) (Dejardin et al., 2014). Defects in the autograft treatment group were treated with corticocancellous autograft (see below). Defects in the negative control group did not receive any additional treatment.

On the day of surgery, dogs were premedicated with acepromazine (0.3 mg/kg), glycopyrrolate (0.01 mg/kg), and hydromorphone (0.1 mg/kg) administered intramuscularly (IM). Anesthesia was induced with propofol (6 mg/kg maximum dose) administered IV to effect. Upon intubation, general anesthesia was maintained with isoflurane (2–4% to effect). Each dog received a morphine epidural (0.1 mg/kg, preservative-free morphine) at the L7-S1 disk space. Perioperative cefazolin (22 mg/kg IV) was administered 30 min prior to skin incision and every 90 min until skin closure. Perioperative monitoring included pulse oximetry, indirect blood pressure, end-tidal CO_2_, and electrocardiography (ECG) at 5 min intervals and body temperature at 15 min intervals. All dogs were warmed with Bair Hugger Model 505 forced air warming blankets (Augustine Medical, Eden Prairie, MN) and received warm lactated ringer’s solution (LRS, 10 ml/kg/h) during anesthesia. Hair was clipped from the right pelvic limb from the dorsal and ventral midlines distally to below the hock. For the dogs in the positive control group, hair was clipped in an 8 cm × 8 cm square centered over both right and left proximal greater tubercles. An initial surgical preparation was performed with three alternating scrubs of 4% chlorhexidine and 0.9% saline. Dogs were transported to the surgical suite and positioned in left lateral recumbence. A second sterile surgical prep was performed with three alternating scrubs of 4% chlorhexidine followed by application of ChloraPrep (2% chlorhexidine, 70% isopropyl alcohol, Becton Dickinson, Franklin Lakes, NJ).

**FIGURE 1:**
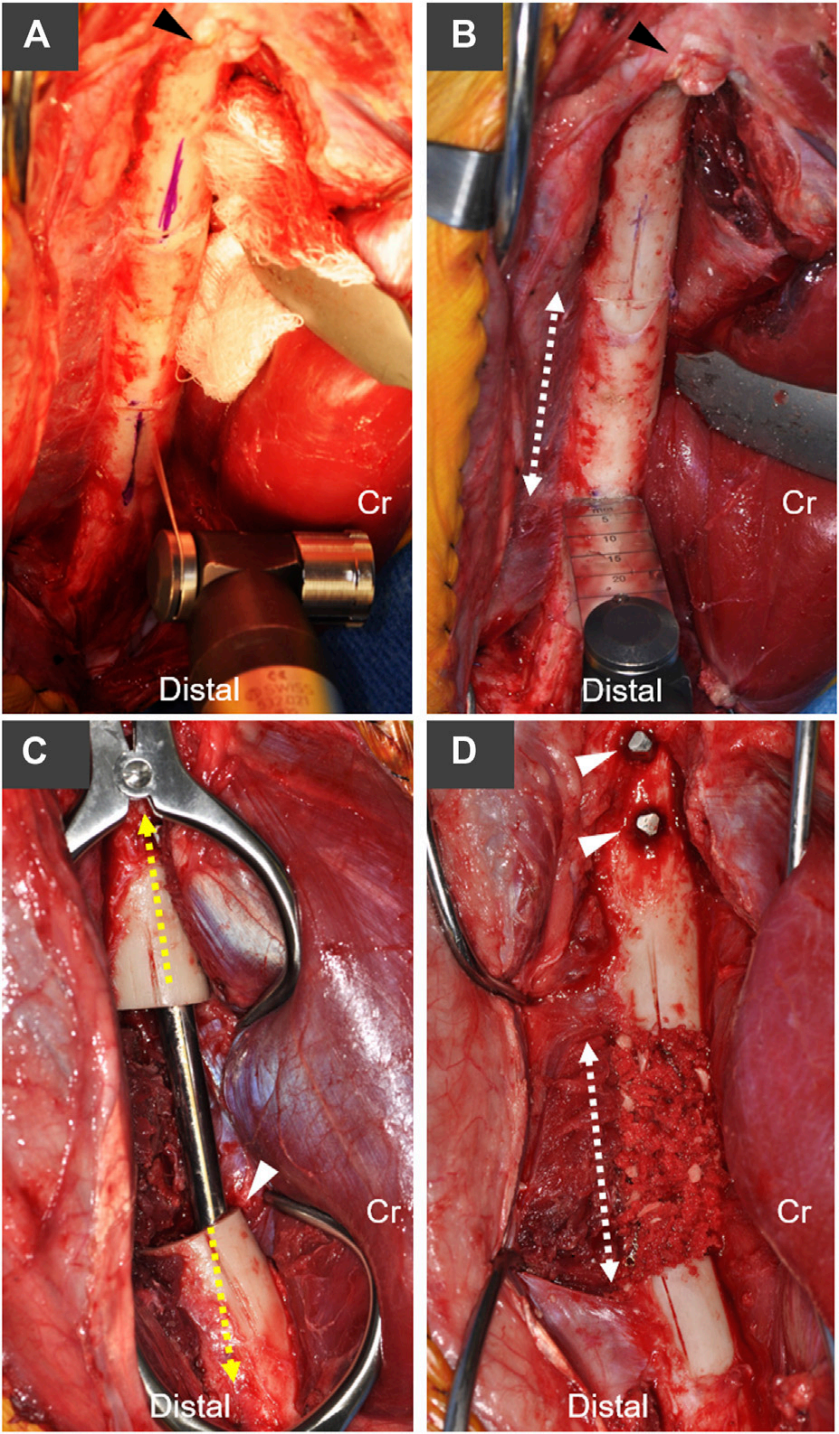
Overview of canine critical-sized femoral defect model with stabilization using an AS-ILN. **(A)** A craniolateral approach to the femur and stifle (knee) joint were performed. Longitudinal index marks were created on the lateral surface of the femur with an oscillating saw and sterile marker to maintain rotational orientation of the proximal and distal bone segments. Arrowhead denotes the base of the greater trochanter, Cr denotes cranial (anterior) position. **(B)** A 4 cm mid-diaphyseal transverse ostectomy was planned (dashed arrow). **(C)** An 8 mm × 172 mm AS-ILN was placed to stabilize the femur (see Figure 2 for specifics of AS-ILN). Dashed line denotes long axis of AS-ILN. Arrowhead denotes the intentional positioning of the cranial (anterior) cortex adjacent to the AS-ILN in order to position the nail more distally in the femoral condyle. Dogs assigned to the negative control group received no additional treatment at the ostectomy. **(D)** Dogs assigned to the autograft treatment group received a 10 cm^3^ cancellous autograft which was supplemented with morselized cortical bone from the ostectomy. Dashed line denotes the extent of the ostectomy and bone graft. Arrowheads denote two proximal locking bolts placed to secure the AS-ILN to the proximal femur.

**FIGURE 2:**
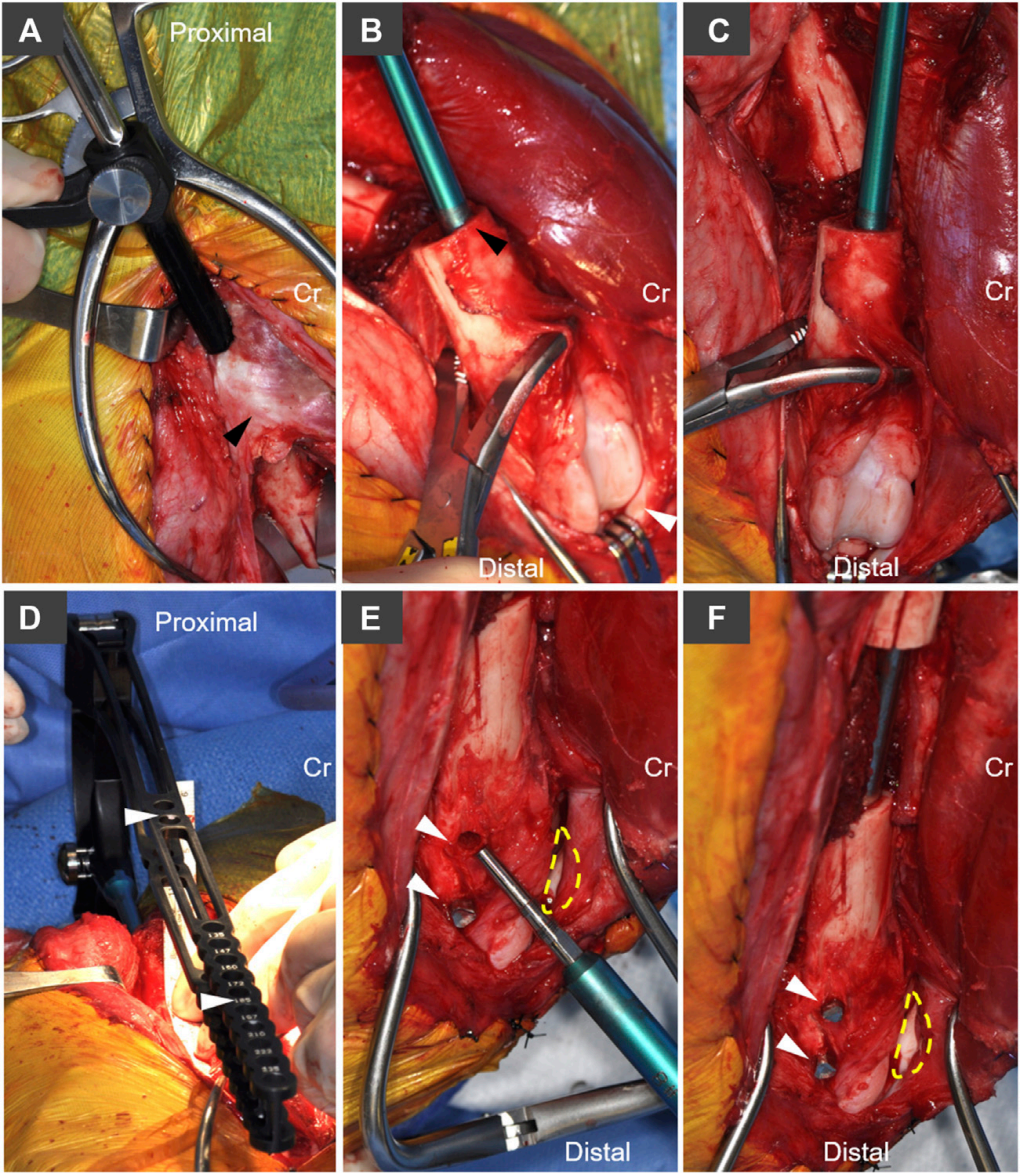
Implantation technique and system-specific instrumentation for the AS-ILN. **(A)** The intertrochanteric fossa was identified and opened with a 1/8 inch (3.2 mm) intramedullary pin. Following pin removal, a system-specific cutting tool and tissue protector were used to enlarge this opening to 8 mm. Cr denotes cranial, arrowhead denotes base of the greater trochanter. **(B)** The distal femur was stabilized with bone holding forceps placed proximal to the trochlea. A system specific cutting tool (8 mm diameter) was used to prepare the cancellous bone bed. To distalize the nail, the cutting tool was intentionally placed adjacent to the cranial (anterior) cortex in the lateral view (black arrowhead). White arrowhead denotes the patella. **(C)** From the cranial (anterior view), care was taken to ensure the cutting tool was oriented parallel to the long axis of the distal femur. **(D)** The 8 mm × 172 mm AS-ILN was attached to an extension and insertion handle and placed in normograde fashion into the femur. A system specific alignment guide (white arrowheads), with cannulations aligned to AS-ILN cannulations, was attached to the handle in order to center drilling through the AS-ILN cannulations for capture by locking bolts. **(E)** Lateral aspect of distal femoral condyle demonstrating placement of angle-stable fixation bolts in drill holes created using the alignment guide in panel **(D)**. Dashed line denotes position of patella. Arrowheads denote bolt holes. **(F)** Final bolt insertion (arrowheads) completes placement of the AS-ILN.

A craniolateral approach to the right femur and stifle (knee) were performed (Johnson, 2014). An oscillating saw [Small Battery Drive System, blade 532.063 (25 × 18 mm), DePuy Synthes, Raynham, MA], was used to create a longitudinal index mark on the lateral surface of the proximal and distal femur adjacent to the proposed ostectomy to ensure anatomic rotational alignment of the femur after ostectomy (Figure 1A). A 4 cm ostectomy, centered on the mid-diaphysis of the femur, was performed with the oscillating saw under continuous saline irrigation (Figure 1B). All periosteum was resected from the ostectomy site. The intertrochanteric fossa was identified and a 1/8 inch (3.2 mm) 316L stainless steel intramedullary pin (Imex Veterinary, Inc., Longview, TX) was used to open the proximal femoral metaphysis. A system-specific cutting awl and tissue protector were aligned with the long axis of the proximal femur and used enlarge this opening to 8 mm (Figure 2A). An 8 mm cutting tool was inserted through this 8 mm opening and into the ostectomy site to create the proximal path for the AS-ILN. The distal diaphysis of the femur was stabilized with clamshell bone holding forceps and the same cutting tool was again used to prepare the path for the AS-ILN into the distal femur (Figure 2B). Care was taken to ensure this path was parallel with the femur in the frontal plane (Figure 2C). In the sagittal plane, the cutting tool was intentionally tipped in a cranioproximal to caudodistal direction to ensure the distal tip of the AS-ILN was located within the caudal aspect of the femoral condyle and directed away from the femoral trochlea (Figure 1C).

A temporary extension and handle were secured to an 8 mm × 172 mm AS-ILN. The 316L surgical stainless steel nail was placed in normograde fashion proximally to distally across the ostectomy site. Nail size and length were selected using pre-operative digital templating (OrthoviewVet, Southampton, Hampshire, United Kingdom). Once in position, the excised 4 cm ring of femoral cortex and a ruler were used to ensure a precise 4 cm mid-diaphyseal defect was present for each femur (Figure 1C). The AS-ILN was secured to the femur using an alignment guide (Figure 2D) and temporary fixation posts in a 2–2 configuration (2 posts proximal, 2 posts distal). Posts were sequentially removed and conical, threaded, locking bolts were cut to length guided by a system-specific depth gauge and locked into the matching threads on the conical AS-ILN cannulations (Figures 2E,F). This coupling created an immediately angle-stable interface thus preventing construct slack (vonPfeil et al., 2005; Dejardin et al., 2006; Lansdowne et al., 2007). After AS-ILN placement, defects were copiously lavaged with saline. Dogs assigned to the autograft treatment group were treated with 10 cm^3^ of autologous cancellous graft harvested from both proximal humeri. Additionally, the 4 cm cortical defect was morselized into 5–8 mm pieces with rongeurs and combined with the cancellous autograft (Figure 1D). Routine, three-layer closure was performed in an identical manner for both groups. The joint capsule was closed with size 0 polydioxanone-II (PDS^®^-II) (Ethicon Inc. Raritan, NJ) in a cruciate suture pattern. Fascia lata was closed with 0 PDS^®^-II in a simple continuous pattern. Subcutaneous tissues were closed with 2–0 poliglecaprone 25 (Monocryl^®^, Ethicon) in a simple continuous manner. Skin was closed with 3–0 Monocryl^®^ in an intradermal pattern.

Post-operative radiographs were obtained (see below) and dogs were recovered from anesthesia in individual runs containing padded bedding. Upon extubation, dogs received carprofen (4 mg/kg SQ once) and hydromorphone (0.1 mg/kg q 6 h) for the first 24 h. Oral carprofen (4 mg/kg q 24 h) was continued for 5 additional days. A single injection of cefovecin sodium (8 mg/kg, Convenia^®^, Zoetis, Parsippany, NJ) was administered subcutaneously for post-operative antibiotic coverage. Surgical incisions and limb use were monitored daily for 5 days post-surgery, then weekly for the remainder of the study. Controlled, slow leash walking of 15-min duration (4 times daily) was initiated 1 day post-surgery. At 6 weeks, leash walks were increased to 30 min (4 times daily) for the duration of the study. At 18 weeks, dogs were humanely euthanized by administration of IV dexmedetomidine and butorphanol (see above) followed by barbiturate overdose (120 mg/kg for the initial 4.5 kg and 60 mg/kg for each additional 4.5 kg).

### Radiography and computed tomography (CT)

Orthogonal femoral radiographs (craniocaudal, mediolateral) were obtained pre-operatively, immediately post-operatively, and at 6, 12, and 18 weeks using a Sound-Eklin Digital Radiography (DR) system (Sound-Eklin, Carlsbad, CA) with a Cannon digital detector plate. Images were stored on a local picture archiving and communication system (PACS) and viewed with a commercially available veterinary viewing software (eFilm 3.0, Merge Healthcare, Milwaukee, WI). At study conclusion, radiographs were exported, blinded to remove animal identity, and randomized in pairs (craniocaudal and mediolateral radiographs) for radiographic scoring by a board-certified veterinary radiologist. An ordinal scoring system was utilized to assess healing as previously described (Faria et al., 2007; Schmiedt et al., 2007). Briefly, craniocaudal and mediolateral radiographs were evaluated on a 1 – 4 scale as follows: 1: ≤1 cortex spanned by bridging callus; 2: 2 cortices with bridging callus; 3: 3 cortices with bridging callus; and 4: bridging callus present on all four cortices.

CT imaging of musculoskeletal tissues containing metal implants can be challenging due to the effects of beam hardening and metal-associated artifacts. Reduction of metal artifacts can be achieved by using specific CT parameters (Berg et al., 2006; Lee et al., 2007). Dogs were imaged with a Siemens SOMATOM Definition AS 40-slice CT immediately post-operatively and again at 6, 12, and 18 weeks. For each scan, the femur was aligned so that the long axis of the gantry and the interlocking nail were coaxial. Sequences were acquired using high kVp (120–140), high mAs (200), low pitch (0.6–0.75), low collimation, thin slices (0.6 mm), with no overlap. For both radiographic and CT imaging, digital imaging and communications in medicine (DICOM) images were stored on a local PACS server with local and off-site image storage.

### Quantitative analysis of CT

Quantitative assessment of CT images was performed using a commercially available segmentation software (Mimics V.19, Materialise NV, Leuven, Belgium). After creation of a project file, the region of interest (ROI) was identified for quantification. Using the analyze tool and the post-operative CT for each dog, two points were placed in the center of the AS-ILN, one at the proximal and one at the distal end of the ostectomy. These points were placed at the location of the first complete cortical ring adjacent to the osteotomy using transverse plane images. Using the distance measuring tool in the sagittal plane, the distance from the proximal end of the AS-ILN to the proximal point and the distance from the distal end of the AS-ILN to the distal point were determined and recorded. The distance tool was also used to confirm 4 cm (40 mm) ostectomy had been performed. These two distances (specific for each femur) were used to identify the location of the proximal and distal points on 6, 12, and 18 weeks CT scans, thus ensuring that identical regions were evaluated throughout the study.

For all scans, a cylinder (height = 40 mm, radius = 15 mm) was created using the analyze/create cylinder tool to further define the ROI. The proximal and distal extent of the cylinder were coplanar with the proximal and distal reference points. Radius of the cylinder was selected such that the ROI would contain an area 1.5x the diameter of the resected femoral diaphysis. Multiple slice editing (set in thresholding mode) was used on transverse plane images to create a new mask for either bone [700–2,000 Hounsfield units (HU)] or both bone and soft tissue (250–2,000 HU) within the ROI. Prior to creation of 3D objects, multiple slice editing (set in remove mode) was used to remove the AS-ILN and beam hardening artifact from each mask using the ellipse tool (width/height = 42). Bone and bone/soft tissue masks were transformed into 3D objects using the calculate part tool and the “optimal” setting. Volume (mm^3^) and surface area (mm^2^) were recorded.

### Mechanical testing

After euthanasia, left and right femurs and associated soft tissues were harvested from the positive control group. Due to the lack of healing in the negative control group, these dogs were not evaluated with mechanical testing. Femurs were placed in 10% neutral buffered formalin (NBF) then stored for 4 weeks prior to analysis. Torsional analysis of non-operated, left and operated, right femurs from the autograft treatment group was performed to compare the mechanical properties of the intact and operated bones and to account for disparate properties of bones between dogs and the effects of 10% NBF fixation (Öhman et al., 2008; Wright et al., 2018). The testing protocol was performed as previously described, with minor modifications (Dejardin et al., 2014). Femurs were cut to 16 cm length, with AS-ILNs remaining in place within the three right femurs. Two divergent brass screws were placed in the ends of each femur, avoiding contact with the AS-ILN (if present) to improve stability during potting. Femurs were potted in polyester body filler (“Fiber Strand” 6371, The Martin Senour Co., Cleveland, OH) in custom designed test fixtures. An alignment guide was used to ensure that each bone was potted so that the AS-ILN was co-axial with the torsional testing axis. For femurs containing an AS-ILN, all locking bolts were removed prior to potting. This ensured that the AS-ILN would not interfere with assessment of mechanical properties during torsional loading. Potting was performed such that the distance between the innermost points of the potting and potting fixtures was identical for all specimens.

The testing protocol was performed with a 135 Nm load cell (5330–1200, Interface Inc. Scottsdale, AZ), rotary encoder (BHW16.05A72000-BP-A, Baumer Electric, Southington, CT) and sevohydraulic testing machine (1331, Instron Corp. Canton, MA) under displacement control (1°/sec) at a sampling rate of 100 Hz to a maximum displacement of 25 of internal rotation. Torsional stiffness, maximum deformation angle at failure, load at failure (Nm), and mode of failure were determined. Torsional stiffness was calculated from torque (Nm) vs angle of twist (º) graphs from 0 to 5. To limit structural damage of the specimens, tests were automatically interrupted upon detection of a 5% drop from peak (failure) torque. Following this test, the potting material and AS-ILN were carefully removed and the spiral fractures were anatomically reconstructed and stabilized with 0 polydioxanone-II suture material. Paired bones were returned to 10% NBF in preparation for histologic analysis. Removal of the AS-ILN and reconstruction of the spiral fracture allowed histologic assessment of femurs evaluated biomechanically, in pursuit of the Principle of the 3R’s of animal research (Kirk, 2018; Angelis et al., 2019).

### Histology

After gross evaluation and photography, the cranial surface of each femur was marked with tissue dye to maintain orientation during processing. The AS-ILNs were removed from the autograft treatment group upon completion of biomechanics (see above). Negative control specimens containing AS-ILNs were imaged with an automated computed tomography machine (X50, North Star Imaging, Rogers, MN, United States) to locate the AS-ILN and center of the femoral defect (non-union). Proximal and distal ends of the femur were transected using a 6-inch trim saw (Hi-Tech Diamond, Westmont, IL, United States) and the AS-ILN was removed. Tissue samples were placed in a 10 to 1 ratio of decalcification solution (Formical-4™, StatLab Medical Products, McKinney, TX, United States) and were agitated for 15 days with solution exchange performed at least once weekly. Samples were removed from decalcification solution, rinsed with deionized water followed by phosphate buffered saline (PBS), and manually sectioned in the sagittal plane from cranial to caudal end. Samples were subdivided into two or three additional segments to fit within jumbo histology cassettes (7.5 × 5.2 × 1.8 cm) and returned to decalcification solution for 45 additional days. Next, samples were rinsed with deionized water and PBS, placed within pre-labelled jumbo cassettes, and processed with an automatic tissue processor (Excelsior AS, Thermo Fisher, Waltham, MA, United States) on a 30-h run with alcohol gradations of 75–100%, clearing reagents (xylenes), and vacuum-mediated paraffin wax infiltration. Samples were embedded (HistoStar Embedding and Cold Module, Thermo Fisher) using a jumbo metal mold. Blocks were sectioned at 5–6 µm onto jumbo slides with a rotary microtome (Microm HM355S, Thermo Fisher) and stained with hematoxylin and eosin (H&E) and Masson’s trichrome. Slides were scanned at 10 × magnification with an Olympus VS.120 Virtual Slide System (Olympus Life Sciences, Waltham, MA, United States). Scanned images were imported into Adobe Photoshop CS6 13.0.0 (Adobe, San Jose, CA, United States), white balanced, and re-assembled to create a cohesive mosaic overview of each femur. Representative histologic mosaics (one femur from each treatment group) were selected for histologic results.

### Statistics

Due to small sample size, both ordinal (radiographic scoring) and continuous (quantitative CT, biomechanics) data were reported as median and range. Descriptive statistics were performed using GraphPad Prism 6.0 (GraphPad Inc. San Diego, CA).

## Results

### Surgical model

Median age for dogs assigned to the autograft group was 5.5 years (range: 3.5–6.5 years) and median body weight was 25.4 kg (range: 23.7–35.8 kg). Median age for dogs assigned to the negative control group was 4.5 years (range: 2.5–6 years) and median body weight was 27.3 kg (range: 25.7–28.6 kg). Incisions healed without complication in all dogs. All dogs were toe-touching on the operated limb the day after surgery, with return to a normal limb use 7–10 days post-operatively. Limb use was maintained for all dogs through the remainder of the study.

### Radiographic assessment of healing

**FIGURE 3:**
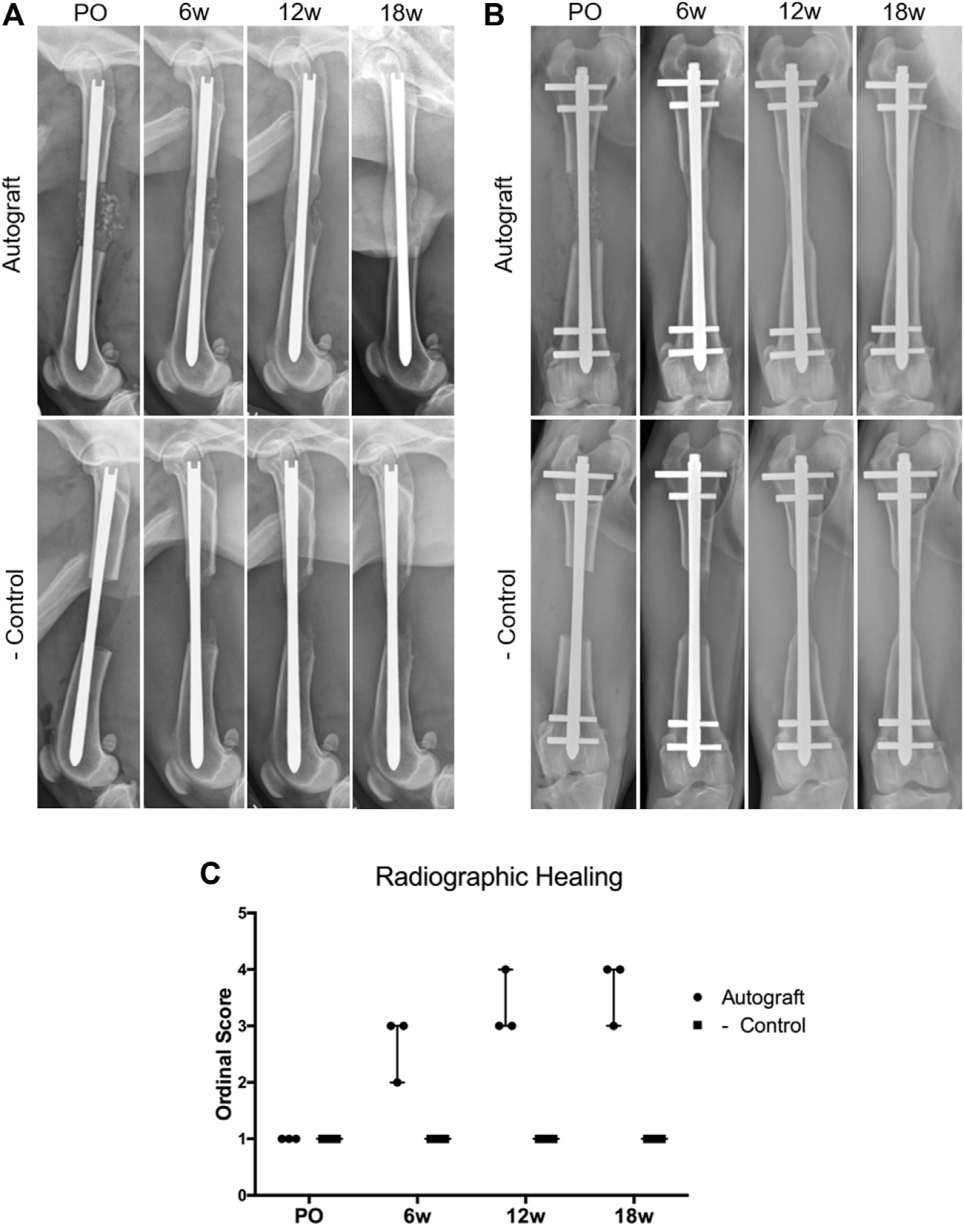
Assessment of critical-sized defect healing by ordinal radiographic scoring. Dogs were radiographed immediately post-operatively (PO) and again at 6, 12, and 18 weeks. **(A)** Representative mediolateral radiographs for the autograft and negative control groups. Note the presence of bone graft in the autograft group, which resulted in smooth, bridging callus by 18 weeks. Callus formation was present in the negative control group at 6,12, and 18 weeks; however, the callus was unable to bridge the critical-sized defect. The AS-ILN implant remained unchanged. **(B)** Representative craniocaudal (anteroposterior) radiographs. Smooth bridging callus developed in the autograft group at 6 weeks and became more substantial at 12 and 18 weeks. Minimal callus formation was identified in the negative control. The AS-ILN implant remained unchanged. **(C)** A previously described ordinal scale was used to score radiographic healing. Orthogonal radiographs from each dog were randomized and evaluated by a board-certified veterinary radiologist blinded to time point, dog, and treatment group. Data were reported using a scatter plot denoting median and range.

Representative radiographs and ordinal scoring data for autograft and negative control groups are provided in Figure 3. On mediolateral (Figure 3A) and craniocaudal (Figure 3B) radiographs, bone graft is visible within the ostectomy site immediately post-operatively in the autograft group. By 6 weeks, bridging callus was visible, which increased in size and opacity at 12 and 18 weeks. This callus enhancement was captured in the ordinal scoring data (Figure 3C). In the negative control group, a small amount of callus formed at 6 weeks. However, callus formation did not progress to bridge the critical-sized defect nor was callus maturation observed. Ordinal scoring data remained at baseline. Importantly, implant complications were not identified in either treatment group.

### Quantification of bone and soft tissue volume via CT

**FIGURE 4:**
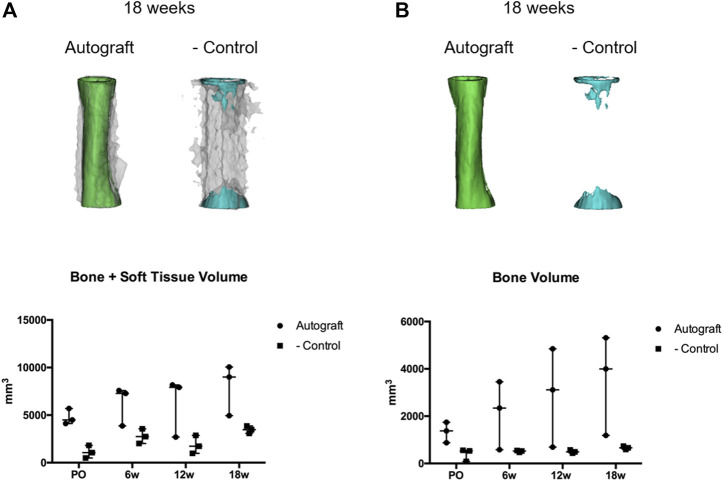
Quantitative assessment of bone and soft tissue using computed tomography (CT) and Mimics segmentation software. CT data from autograft and negative control dogs were exported to a commercially available segmentation software (Mimics 19.0, Materialise Inc., Leuven, Belgium). Segmentation of bone and soft tissues were performed to generate 3D models and quantify tissue within the critical defects. **(A)** Representative bone within autograft (green) or negative control (blue) treatment groups at 18 weeks. Soft tissue is represented by the translucent gray overlay. **(B)** Representative bone within autograft (green) and negative control (blue) treatment groups at 18 weeks. Volumetric data (mm^3^) for bone + soft tissue **(A)** or bone **(B)** were reported using scatter plots denoting median and range.

Representative bone/soft tissue reconstructions with bone volume data are provided in Figure 4. In the autograft group (green), a complete cylinder of bone and soft tissue (gray) callus was present, bridging the entirety of the critical-sized defect as shown in representative 18 weeks reconstructions (Figure 4A). Bone and soft tissue callus volume increased over time in this group. Interestingly, one of the dogs in the autograft group (age 6.5 years), exhibited a reduced healing response when compared to the other dogs within this group. In the negative control group (blue), callus formation occurred but failed to bridge the critical-sized defect as shown in representative 18 weeks reconstructions. Defects within the negative control group were primarily filled with soft tissue. Bone and soft tissue callus quantification for the negative control group remained low throughout the study.

### Mechanical testing of autograft treatment group

**FIGURE 5:**
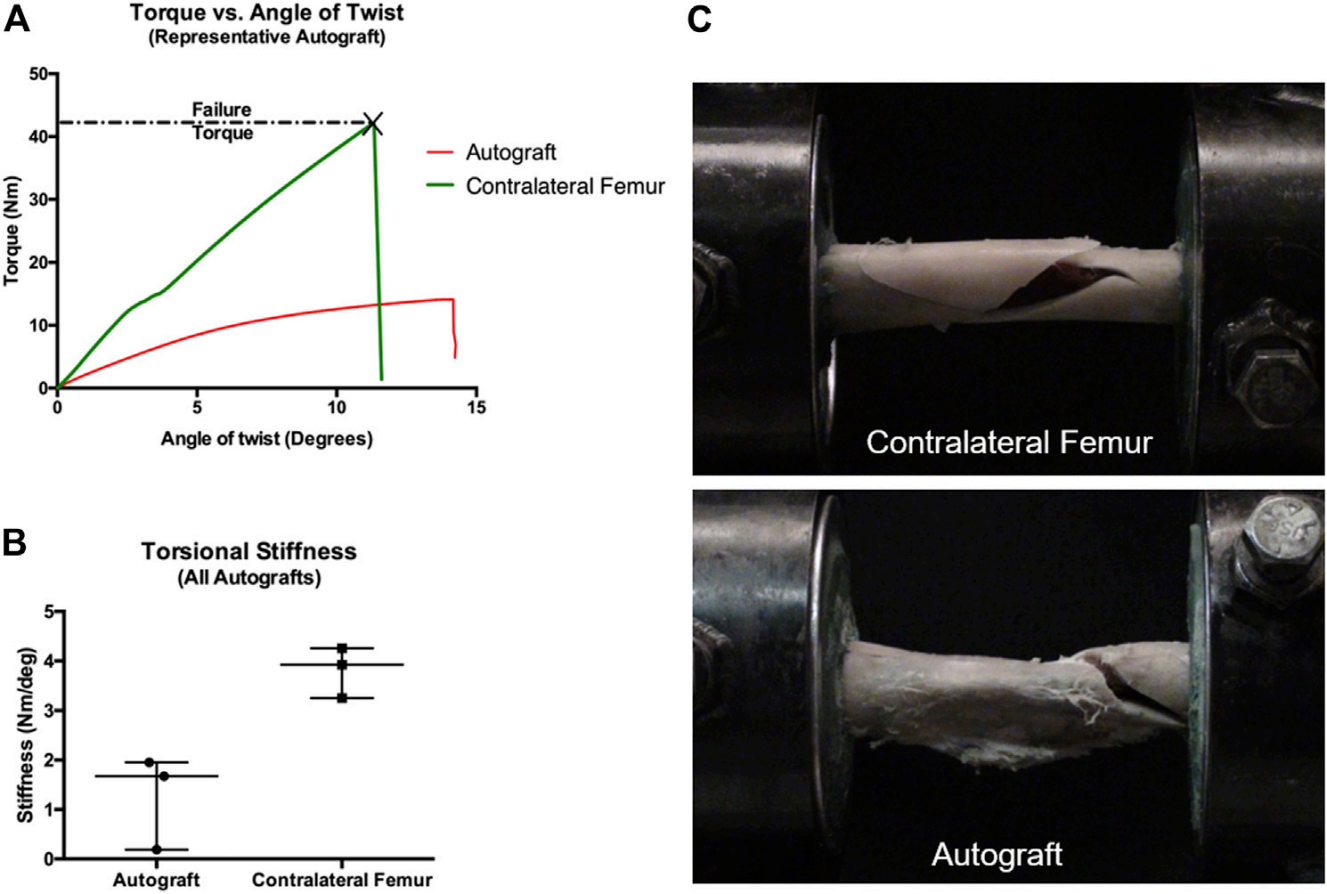
Mechanical testing of paired femurs (operated and contralateral non-operated) from autograft treatment group. For femurs containing an AS-ILN, the implant was identified within the proximal femur and a custom alignment device was used to identify the long axis of the AS-ILN and to position the AS-ILN in the center of the torsional testing axis. Prior to potting, locking bolts were removed. Specimens were potted such that the distance between innermost points of the potting was identical for all specimens. **(A)** Representative load-deformation (torque-angle of twist) graph for an autograft femur (red) and contralateral, non-operated femur (green). **(B)** Torsional stiffness values (Nm/^0^) were determined from the load-deformation curves from 0 to 5 and reported as scatter plots denoting median and range. **(C)** Representative mode of failure images. Contralateral, non-operated femurs failed by spiral fracture with comminution. Critical-sized defect femurs treated with autograft failed by spiral fracture through the callus at the junction with the distal diaphysis.

Paired femurs from the autograft group were assessed for mechanical properties under torsional loads. The negative control group was not assessed due to the fact that none of the femurs developed bridging callus. Torsional mechanical testing results are reported in Table 1; Figure 5. Not unexpectedly, the properties of operated femurs were lower than that of the contralateral, non-operated femurs. A representative load-deformation curve is provided in Figure 5A, with torsional stiffness data for all bones displayed in Figure 5B. Importantly, one operated femur exhibited low torsional stiffness. This was the femur from the autograft group with the lowest amount of callus volume (Figure 4). In the operated femurs, failure occurred by spiral fracture through the callus or at the junction of the callus and distal diaphysis. In contralateral, non-operated femurs, a similar fracture pattern and location was noted, although with some degree of comminution (Figure 5C).

**TABLE 1:** Biomechanical properties (torsion) of autograft treatment group.

	Stiffness (Nm/°)		Max def at failure (°)		Torque at failure (nm)		Failure mode
	Median	Range	Median	Range	Median	Range	(Number)
Autograft	0.19	0.19–1.67	7.0	7.0–14.0	12.0	1.7–14.0	Spiral fracture through callus (2) or junction of callus and distal bone (1)
Contralateral femur	3.92	3.25–4.26	12.5	11.3–12.8	42.0	34.0–54.0	Spiral fracture with comminution (3)

Table 1: At 18 weeks, three pair of femur from the autograft treatment group (*n* = 6 femur) were assessed for biomechanical properties in torsion. Autograft = Right femur, 4 cm defect, stabilized with AS-ILN, corticocancellous autograft. Contralateral femur = left femur, non-operated limb. Abbreviations: Nm/° = Nm per degree; Max Def at Failure (°) = Maximum angle of deformation at failure in degrees.

### Histologic assessment of bone healing

**FIGURE 6:**
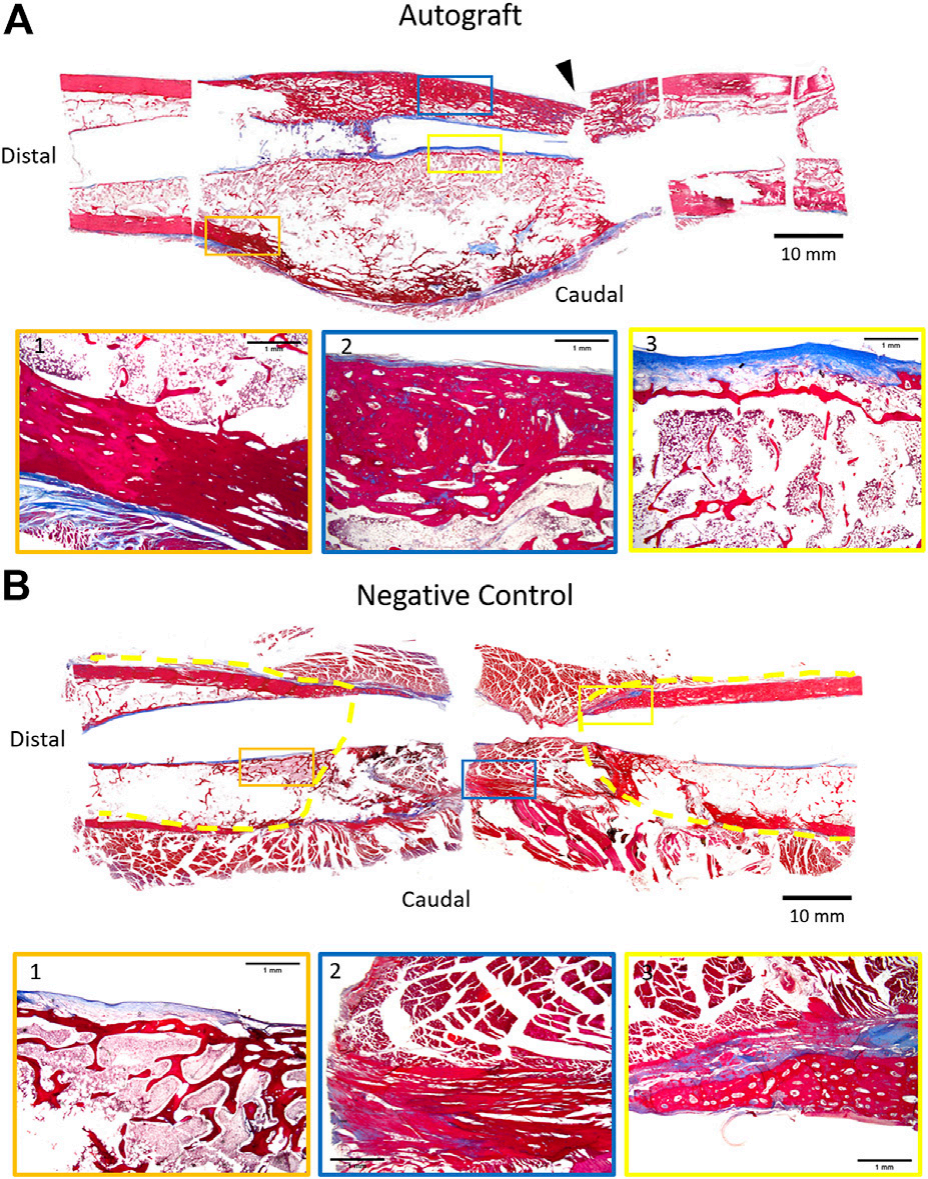
Representative histology of autograft and negative control at 18 weeks. Upon completion of mechanical testing (autograft), femurs were carefully removed from potting. AS-ILNs were removed and the fractured segments were temporarily stabilized with circumferential sutures. Samples were photographed, marked to document spatial orientation, and decalcified. To provide a comprehensive histologic image of the 4 cm defect, specimens were sub-sectioned to fit within jumbo cassettes and processed for histology. Sections were stained with Masson’s Trichrome, followed by scanning and digital reconstruction to generate a low magnification digital mosaic. **(A)** Representative histology of femur treated with autograft. Arrowhead denotes location of mechanical testing failure point. The cortical region at mid-diaphysis is expanded by trabecular bone (Insets 1,2) admixed with fibrovascular connective tissue (Inset 3). **(B)** Representative histology of negative control femur. Dashed yellow lines denote extent of callus formation. In the negative control, the mid-shaft cortical region was devoid of trabecular bone and was filled by fibrovascular connective tissue (Inset 2) consistent with a non-union fracture. For low magnification montages, bar = 10 mm. For insets, bar = 1 mm.

Representative histology of autograft and negative control femurs are provided in Figure 6. It is important to note that for the autograft treatment group, histology was performed on the same group of femurs that were assessed biomechanically. In the autograft group, the mid-shaft cortical region was markedly expanded by an orderly layer of trabecular bone (Figure 6A, insets 1,2) admixed with a moderate amount of fibrovascular connective tissue and regions containing typical bone marrow. Regions of moderate endochondral ossification extended through the fibrous connective tissues and woven bone. Of note, the central medullary region in contact with the AS-ILN exhibited an orderly fibroblastic cell population within an eosinophilic matrix consistent with collagen (Figure 6A, inset 3). For the negative control, the mid-shaft cortical region was disrupted and filled with the fibrovascular connective tissue of a non-union fracture (Figure 6B, inset 2). Endochondral ossification was present within the fibrous connective tissues adjacent to the fracture (Figure 6B, inset 3) but, consistent with radiography and CT, did not bridge the critically-sized defect. Similar tissue and cell architecture was noted adjacent to the AS-ILN (Figure 6B, inset 1).

## Discussion

The results of this study support both of our hypotheses. In the presence of a critical-sized canine femoral defect, the AS-ILN provided sufficient and consistent construct stability for the duration of the study. There were no complications and dogs quickly began using the operated limb with subsequent resolution of visible lameness. Dogs assigned to the autograft group experienced early bridging of the critical-sized defect as early as 6 weeks, with continued bone healing to the 18 weeks end-point. Dogs assigned to the negative control group ambulated without complication despite no radiographic or CT evidence of bone healing.

Translation of promising bone tissue engineering treatments for critical-sized bone defects from conception to successful clinical application is a difficult task and has been referred to as the “valley of death” for product development (Reichert et al., 2010; Sastry, 2014; Zheng et al., 2017). Due to the regulatory classification of many tissue engineering scaffolds as medical devices (Sastry, 2014; Zheng et al., 2017), it is unknown how many tissue engineering devices have failed to translate to the clinical setting. In pharmaceutical development, it has been reported that only 11% of new drugs successfully move through Phase I-III clinical trials and not all of those proceed to market (Wong et al., 2019). It is clear that novel bone tissue engineering devices experience even lower levels of success. Despite decades of intense effort, thousands of peer-reviewed publications, and hundreds of millions of research dollars, an approved tissue engineering device for critical-sized long bone defects is currently not available (Hollister and Murphy, 2011; Amini et al., 2012).

Novel bone tissue engineering devices must be rigorously evaluated prior to initiation of clinical trials in human beings. Large animal species such as the sheep, pig, goat, and dog are often used for translational studies during product development. When used properly, large animal models are highly useful for determining scalability, developing successful workflow, obtaining preliminary safety and efficacy data, and identifying benchmarks for success during clinical trials (Liebschner, 2004; Horner et al., 2010; Muschler et al., 2010; Ribitsch et al., 2020). As detailed in our introduction, the dog represents a strong translational model for bone tissue engineering studies. Presently, only a handful of studies have described canine critical-sized or non-union defect models (Volpon, 1994; Markel et al., 1995; Arinzeh et al., 2003; Lindsey et al., 2006). These describe critical-sized defects of the canine radius, femur, and tibia and rely on diverse stabilization methods such interlocking nails, bone plates, and external fixation. Thus, there is no clear consensus on the ideal canine critical-sized defect model to evaluate bone tissue engineering devices, nor has there been much focus on refinement and reduction of the canine model in pursuit of the Principle of the 3R’s (Kirk, 2018; Angelis et al., 2019). Our goal was to develop a refined canine critical-sized defect model and report detailed methods for both surgical technique and comprehensive outcome measures in pursuit of the Principles of the 3R’s.

In the present study, we successfully refined a canine critical-sized femoral defect model using an AS-ILN designed to eliminate acute angular deformation (slack) and provide consistent biomechanical performance across all animals, regardless of the assigned treatment group. The AS-ILN used in the present study has been shown to reduce lameness, lead to consistent and accelerated bone healing, and produce superior callus mechanical properties when compared to a traditional interlocking nail (Dejardin et al., 2014). These advances are due to unique features of the AS-ILN system such as the conical, threaded locking mechanisms of fixation bolts and the hourglass profile of the AS-ILN (Dejardin et al., 2012). Additionally, when compared to a traditional veterinary interlocking nail in mechanical tests, the AS-ILN provided more consistent angular deformation in torsion, eliminated construct slack (Dejardin et al., 2006), responded more uniformly to bending loads (Dejardin et al., 2009), and produced similar compliance regardless of defect size (Ting et al., 2009).

The AS-ILN was implanted without complications using surgical techniques similar to those used in human beings. Due to its unique biomechanical properties and position within the medullary canal, the AS-ILN allowed for a rapid return to limb use, with all dogs ambulating normally as early as 7–10 days post-operatively. The AS-ILN provided excellent clinical function over an extended period of time, even for dogs assigned to the negative control group. This is highly relevant as many bone tissue engineering studies require a negative control group wherein the implant system must provide sufficient stability for normal ambulation in the absence of healing. Additionally, a small AS-ILN system has also been developed and is in clinical use (Marturello et al., 2020, 2021). This is highly relevant for investigators that utilize rabbit critical-sized defect models for bone tissue engineering studies. An AS-ILN system was not previously available for use in this species. The authors suggest that the small AS-ILN is well suited for rabbit femoral or tibial critical-sized defect studies.

Due to the location of the AS-ILN within the central axis of the bone, the implant system did not obstruct radiographic scoring of cortical bridging (Figure 3). The relatively small cross-sectional area of the AS-ILN, located centrally within the bone, facilitated quantification of both soft and hard callus when paired with CT scanning parameters designed to minimize metal artifact (Figure 4). Moreover, the AS-ILN served as reference point to precisely identify the region of interest (ROI) for each dog and ensured that tissue quantification was performed at identical locations and over multiple time points. The location of the AS-ILN within the central axis of the bone facilitated mechanical testing and histological assessment of callus formation. The AS-ILN was used to identify the long axis of the femur, which was positioned along the central axis of the materials testing machine for torsional testing. Upon removal of distal fixation bolts and fixation of bones in the test fixtures, the AS-ILN did not interfere with torsional testing similar to prior work (Dejardin et al., 2014). The resulting mechanical testing data were thus representative of the fracture callus. Finally, the testing protocol was developed such that once ultimate failure occurred, loading was immediately stopped in order to allow reconstruction of the bone column for histological assessment. This testing protocol was designed to optimize objective evaluation of bone healing using various outcome measures while reducing sample size (animal numbers).

Much consideration was given to the decision to perform histology on formalin-fixed, decalcified tissues that, in the case of the autograft group, had been mechanically tested. The gold standard technique for histologic assessment of orthopedic implants and adjacent tissues involves methyl methacrylate (i.e. “plastic”) embedding and non-decalcified sectioning (Maglio et al., 2020). Methacrylate embedded sections are essential for evaluation of the implant/tissue interface of orthopedic implants and bone. The AS-ILN used in the present study is composed of 316L surgical stainless steel, which is a common, biocompatible alloy for orthopedic implants (Syrett and Davis, 1979; Erdmann et al., 2010). Moreover, the objective of this study was to use an existing AS-ILN to refine and reduce animal numbers, not to perform a detailed histologic analysis or histomorphometry of a novel implant and adjacent tissues. For this reason, histology was considered complimentary to the quantified CT results. Given this reasoning, it was not justifiable to operate additional cohorts of dogs for methacrylate-embedded, non-decalcified histology. Should the techniques detailed in the present study be used in the future to evaluate novel biomaterials for bone tissue regeneration, additional cohorts of dogs might be necessary for methacrylate embedded histology to meet regulatory criteria and allow a detailed evaluation of the interactions between device and adjacent host tissues.

While limitations are expected with any study, our goal was to describe a clinically-relevant large animal translational model for future bone tissue engineering studies. First, although skeletally mature hounds were used, dog age was somewhat variable (range: 2.5–6.5 years). The oldest dog assigned to the autograft group (6.5 years) produced the lowest amount of callus (Figure 4), which correlated to low torsional stiffness and torque at failure (Figure 5; Table 1). While age is the likely explanation for the reduced healing response in this dog, it is possible (although not plausible based on prior studies) that the AS-ILN provided disparate biomechanical stability in this animal. It is well known that bone healing is more robust in younger animals (Risselada et al., 2005; Mehta et al., 2010). A study in canine clinical patients documented more rapid healing in younger dogs (Averill et al., 1999). To definitively address the issue of age on bone healing in this model, an additional canine critical-sized defect study would be necessary with closely controlled ages (young cohort versus old cohort). Interestingly, older dogs may prove useful in this model in order to more rigorously challenge autograft control groups, novel bone healing agents, or new tissue engineering devices.

Additionally, all dogs in the present study were intact males due to subject availability at the time the study was initiated. While sex has been shown to effect bone healing in rodent induced-injury models (Mehta et al., 2011; Haffner-Luntzer et al., 2021), in the canine clinical setting the AS-ILN has been successfully placed in both intact and sterilized male and female dogs and cats with no detectible effect of subject sex (Marturello et al., 2021). Future studies may be warranted to determine whether sex or sterilization status has an effect on bone healing in this canine experimentally-induced critically-sized femoral defect model.

Second, we elected to create critical-sized defects via surgical ostectomy rather than traumatic fracture. This decision was made for ethical reasons to reduce morbidity, as well as to reduce inter-animal variability and maintain small animal numbers. Third, while segmentation of CT is an accepted method for quantifying bone volume and surface area in large animal models (Dejardin et al., 2014), CT is unable to assess bone mineral density (Robertson et al., 2017; Booz et al., 2020). Dual x-ray absorptiometry (DXA) would be required to determine bone mineral density during healing in this canine critically-sized femoral defect model. Fourth, the mechanical testing protocol was limited to torsional loading on formalin-fixed bone. While determining the biomechanical properties of fracture callus in the autograft-treated femurs under both bending and torsional loads could have generated useful data, torsion has been shown to be the major loading mode of long bones (Gautier et al., 2000; Yang et al., 2014). Furthermore, the presence of the AS-ILN within the medullary cavity would have severely masked the contribution of the fracture callus to bending stiffness. Lastly, regarding the mechanical testing of formalin-fixed bone, fixation was performed to prepare specimens for routine decalcification and paraffin embedded histology. While concerns have been raised about the impact of formalin fixation on biomechanical testing of long bones, contralateral (non-operated) control femurs were tested in tandem to account for any potential effect of fixation. This concern has been recently drawn into question, it was reported that formalin fixation had no effect on the mechanical properties of human femurs, and that patient-specific associations such as age and gender were retrievable despite formalin fixation (Wright et al., 2018). Another study reported no differences in yield stress, ultimate stress, and tissue hardness for fresh bone or bone stored in 4% formalin for up to 8 weeks (Öhman et al., 2008).

In summary, critical-sized long bone defects represent a tremendous clinical challenge. Although treatments exist, none is uniformly effective. While tissue engineering devices hold much promise for these injuries, a tissue engineering solution has yet to be successfully brought to market. The dog is an excellent large animal model species for translational evaluation of encouraging tissue engineering devices. However, a consensus on the preferred technique for inducing and assessing a canine critical-sized long bone defect model is lacking. This study describes the use of an AS-ILN with superior biomechanical properties to refine the canine critical-sized femoral defect model. Animal numbers were minimized by performing radiographic scoring, quantitative CT, torsional mechanical testing, and histology on a single cohort of dogs randomized to either autograft or negative control treatment groups. This work represents an important advancement for the canine translational model and collectively advances the field toward the Principle of the 3R’s.

## Data Availability

The raw data supporting the conclusion of this article will be made available by the authors, without undue reservation.

## Abbreviations

IV, intravenously; AS-ILN, angle-stabile interlocking nail; SQ, subcutaneous; 3D, three-dimensional; HU, Hounsfield units; ROI, region of interest; DR, digital radiography; PACS, picture archiving and communications system; NBF, neutral buffered formalin; PBS, phosphate buffered saline; CT, computed tomography; DICOM, digital imaging and communication in medicine.
